# Deep Count: Fruit Counting Based on Deep Simulated Learning

**DOI:** 10.3390/s17040905

**Published:** 2017-04-20

**Authors:** Maryam Rahnemoonfar, Clay Sheppard

**Affiliations:** Department of Computer Science, Texas A&M University-Corpus Christi, Corpus Christi, TX 78412, USA; csheppard1@islander.tamucc.edu

**Keywords:** deep learning, agricultural sensors, simulated learning, yield estimation

## Abstract

Recent years have witnessed significant advancement in computer vision research based on deep learning. Success of these tasks largely depends on the availability of a large amount of training samples. Labeling the training samples is an expensive process. In this paper, we present a simulated deep convolutional neural network for yield estimation. Knowing the exact number of fruits, flowers, and trees helps farmers to make better decisions on cultivation practices, plant disease prevention, and the size of harvest labor force. The current practice of yield estimation based on the manual counting of fruits or flowers by workers is a very time consuming and expensive process and it is not practical for big fields. Automatic yield estimation based on robotic agriculture provides a viable solution in this regard. Our network is trained entirely on synthetic data and tested on real data. To capture features on multiple scales, we used a modified version of the Inception-ResNet architecture. Our algorithm counts efficiently even if fruits are under shadow, occluded by foliage, branches, or if there is some degree of overlap amongst fruits. Experimental results show a 91% average test accuracy on real images and 93% on synthetic images.

## 1. Introduction

Recent years have witnessed enormous advancement in the computer vision research based on deep learning. A variety of vision-based tasks, such as object recognition [1,2,3,4,5,6], classification [7,8,9], and counting [10,11,12], can achieve high accuracy. Success of these advanced tasks largely depend on the availability of a large amount of training samples. Labeling the training samples is an expensive process both in terms of time and money. Generating synthetic data for training can provide an alternative solution. One of the objectives of this research is to reduce the overhead of labeling the training samples for object counting problem by creating synthetic dataset for training. This research achieves a fast yield estimation based on deep simulated learning. Accurate yield prediction helps farmers to improve their crop quality. Moreover, it helps in reducing the operational cost by making better decisions on the intensity of crop harvesting and the labor required. 

**Figure 1:**
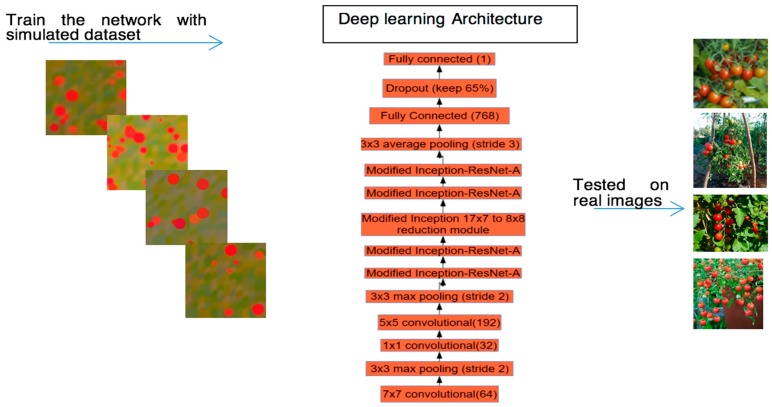
The framework of our research.

There are various challenges faced by computer vision algorithms for counting fruits for yield estimation, namely, illumination variance, and occlusion by foliage, varied degree of overlap amongst fruits, fruits under shadow, and the scale variation. To address these challenges, in this research we developed a novel deep learning architecture which counts objects without detecting them. Our method estimates the number of objects explicitly from the glance of the entire image. In this way, it reduces the overhead of object detection and localization. Our network consists of several convolution and pooling layers in addition to modified Inception-ResNet. The modified version of the Inception-ResNet helps us to capture features in multiple scales. The framework of our approach is depicted in Figure 1. 

The main advantage of this work is that thousands of annotated data on real images are not necessary for training. The network was trained using synthetic images and tested on real images and it works efficiently with 91% accuracy on real images. The proposed methodology works efficiently even if there is illumination variance in the images. 

The following are the contributions of this work:

A novel deep learning architecture for counting fruits based on convolutional neural networks (CNN) and a modified version of Inception-ResNet is presented.

We developed a simulation-based learning method, which is trained on simulated data but tested on real data.

Our approach is robust to occlusion, variation in illumination and scale.

Our algorithm works in less than a second, which is enough to be useful for real-time application.

## 2. Related Work

In typical image classification process the task is to specify the presence or absence of an object but counting problem one requires to reason how many instances of an object are present in the scene. The counting problem arises in several real-world applications, such as cell counting in microscopic images [13], wildlife counting in aerial images [14], fish counting [15], and crowd monitoring [16] in surveillance systems. The method proposed by Kim et al. [17] detects and tracks moving people with the help of a fixed single camera. Later, Lempitsky et al. [18] proposed a new supervised learning framework for visual object counting tasks that optimizes the loss based on the MESA-distance during the learning. Recently, Giuffrida et al. [19] proposed a learning-based approach for counting leaves in rosette (model) plants. They used a supervised regression model to relate image-based descriptors which are learned in an unsupervised fashion to leaf counts.

The current practice of yield estimation based on the manual counting of fruits or flowers by workers is very time consuming and expensive process and it is not practical for large fields. Automatic yield estimation based on robotic agriculture provides a viable solution in this regard. A widely adopted solution for automatic yield estimation is to count fruits or calculate the density of flowers on images using computer vision algorithms [20,21,22,23,24,25,26]. Computer vision-based crop yield estimation methods can be divided roughly into two categories: (1) region- or area-based methods and, (2) counting-based methods. In the literature, there is an ample amount of work dealing with region-based methods [20,21,22,23,24,25,26]. Wang et al. [20] developed a stereo camera automatic crop yield estimation system for apple orchards. They captured images at nighttime to reduce the unpredictable natural illumination in the daytime. Li et al. [21] developed an in-field cotton detection system based on region-based semantic image segmentation. Lu et al. [22] developed region-based color modeling for joint crop and maize tassel segmentation. Despite a wide attention to region-based methods very scarce attention has been paid to counting-based yield estimation methods [27,28]. Linker et al. [27] used color images to estimate the number of apples acquired in orchards under natural illumination. The drawbacks are direct illumination and color saturation, due to which a large number of false positives were observed. Tabb et al. [28] developed a method to segment apple fruit from video using background modeling. 

Recently, deep learning-based object counting methods are gaining popularity. Seguí et al. [10] explored the task of counting occurrences of a concept of interest with CNN. Xie et al. [13] developed a convolutional regression network-based microscopy cell counting framework. Zhang et al. [11] developed cross-scene crowd counting framework based on deep convolutional neural networks. French et al. [29] also explored CNN for counting fish in a fisheries surveillance video. Several authors explored deep learning approaches for fruit/plant detection and recognition [30,31,32,33,34]. To the best of our knowledge, there are no papers related to fruit counting based on deep simulated learning; all of the deep learning-based counting methods rely on object detection and then count the detected instances. Our method estimates the count of objects explicitly from the glance of the entire image. In this way, it reduces the overhead of object detection and localization and it learns explicitly to count. Moreover, the aforementioned techniques are dependent on a large set of labeled data. Labeling the training samples is expensive both in terms of time and money. Here we are generating synthetic data to reduce the overhead of labeling the training samples for object counting problems. Although trained on synthetic data, our method performs very well on real data. 

## 3. Methodology

### 3.1. Synthetic Image Generation

**Figure 2:**
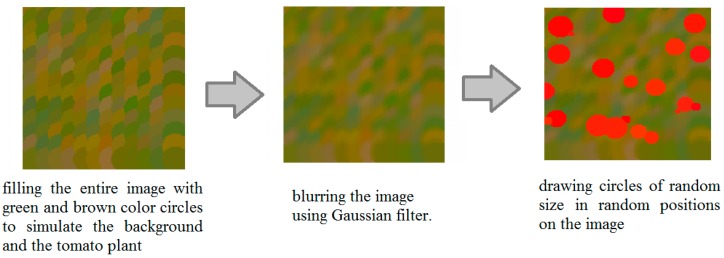
Synthetic image generation.

Deep learning requires large datasets that are time consuming to collect and annotate. To solve this issue, we generated synthetic data to train our network. The training parameters were then tested on real images. The synthetic images were generated as follows. A blank image of size 128 × 128 pixels is created followed by filling the entire image with green and brown colored circles to simulate the background and the tomato plant, which are later blurred by a Gaussian filter. To create the variable-sized tomatoes in the image, several circles of random size are drawn in random positions on the image. Twenty-four thousand images were generated for the training set, and 2400 for the test set. Figure 2 shows the process of generating the synthetic images that were used to train the network. Synthetic tomato images were generated with some degree of overlap along with variation in size, scale, and illumination in order to incorporate the possible complexities in real tomato images.

### 3.2. Convolutional Neural Network

CNN is one of the most notable deep learning approaches; it comprises various convolutional and pooling (subsampling) layers that resembles human visual system [35]. Generally, image data is fed to the CNN that constitute an input layer and produces a vector of reasonably distinct features associated to object classes in the form of an output layer. Between input and output layers there are hidden layers in the form of series of convolution and pooling layers followed by fully-connected layers [36]. The training of the network is performed in forward and backward stages based on the prediction output and labeled ground-truth. In the backpropagation stage, the gradients of each parameter is computed based on the loss cost. All of the parameters will be updated based on the gradients and are updated for the next forward computation. The network learning can be stopped after sufficient iterations of forward and backward stages.

### 3.3. Inception Architecture

While a convolutional layer attempts to learn to filter simultaneously with two spatial dimensions and a channel dimension in a 3D space, the Inception model makes this process easier and, therefore, it empirically appears to be capable of learning richer representations with less parameters. The Inception model would independently look at cross-channel correlations and at spatial correlations. Inception architecture, introduced by Szegedy et al. (Inception-v1) [37], later refined as Inception-v2 [38], Inception-v3 [39] and, most recently, as Inception-ResNet [6], has been one of the best-performing families of models on the ImageNet dataset [40]. Inspired by the success of these models in ImageNet competition, we combined the modified version of Inception-ResNet-A in our CNN network. 

### 3.4. Description of Our Network Architecture

**Figure 3:**
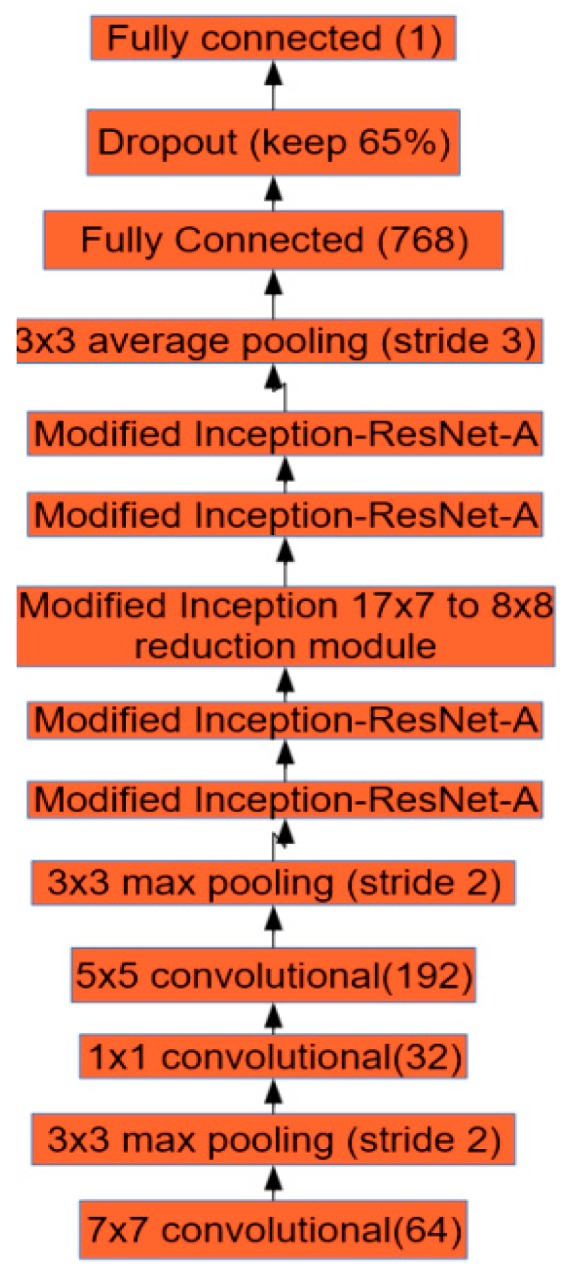
The architecture of our network.

The neural network design for this research is shown in Figure 3. The first layer of the network is 7 × 7 convolution layer followed by 3 × 3 max pooling layer with stride 2. This convolutional layer maps the 3 bands (RGB) in the input image to 64 feature maps using a 7 × 7 kernel function. This condenses information in the network. Reducing the dimensions of the image reduces computation time and allows the model to fit into the GPU’s memory [41]. Similarly, in order to reduce the dimensionality of the feature maps, a 1 × 1 convolution layer is used before another convolution layer of kernel size 5 × 5. 

**Figure 4:**
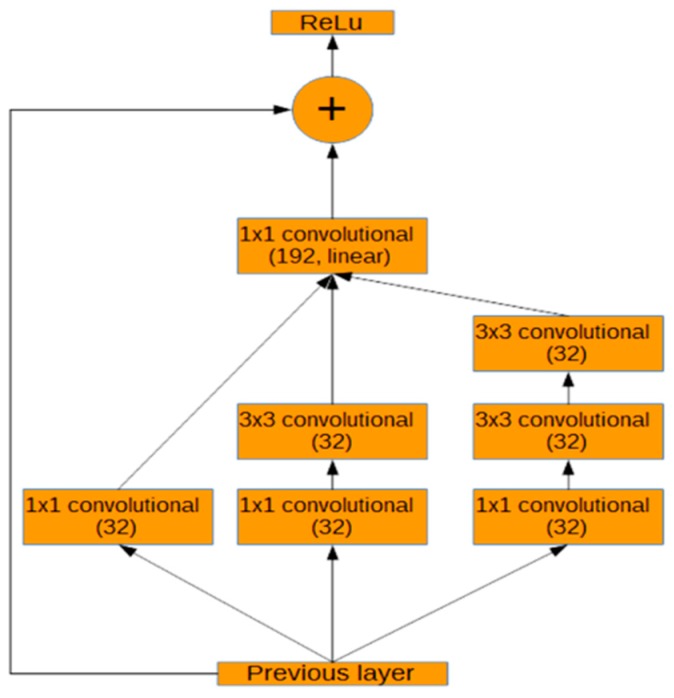
Modified Inception-ResNet-A module.

The size of the objects in the images varies, so an architecture that can capture features at multiple scales is required. For this purpose, we modified the Inception-ResNet-A [6] layer. Two modified Inception-ResNet-A layers follow the normal convolutional layers. Inception-ResNet combines the ideas of Inception, which captures features at multiple sizes by concatenating the results of convolutional layers with different kernel sizes, and residual networks [3], which use skip connections to create a simple path for information to flow throughout a neural network. This architecture was used because of its high performance on several competitive image recognition challenges [6]. Residual networks converge faster because residual connections speed up training in deep networks [3]. Figure 4 shows the design of the modified Inception-ResNet-A module that is used in this work. The final 1 × 1 convolution only calculates 192 features, compared to 256 in the original Inception-ResNet-A [6]. 

**Figure 5:**
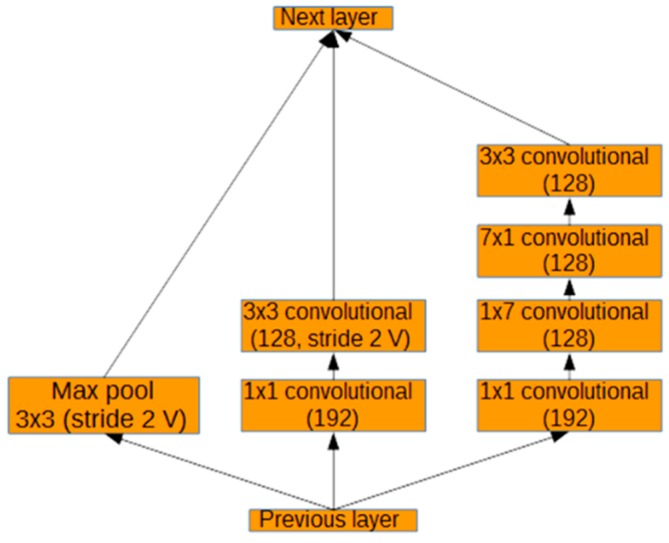
Modified reduction module.

As can be seen in Figure 4, the modified Inception-ResNet-A module consists of three parallel layers concatenated into one. The result of this concatenation is then added to the activations of the previous layer and passed through the rectified linear function. After the modified Inception-ResNet-A layers, a modified Inception reduction module, shown in Figure 5, is used to simultaneously reduce the image size and expand the number of filters. As can be seen in Figure 5, three parallel branches are concatenated into one output. These branches include maximum pooling and strided convolutions without padding (stride 2 V in Figure 5). The middle branch of the module was reduced from an output size of 192 to 128. The right branch of the module was reduced to 192, 128, 128, and 128 output sizes from 256, 256, 320, and 320, respectively. These changes were made in order to fit the network more closely to the complexity of the problem. Before these modifications, the model tended to overfit to the training data and performed poorly on real data. 

After the modified reduction module another set of two Inception-ResNet-A layers were applied, followed by 3 × 3 average pooling because average pooling has been found to improve the accuracy when used before the final fully connected layer [42]. As can be seen in Figure 3 the size of the final fully connected layer is 768. Although deep neural nets with a large number of parameters are very powerful machine learning systems, overfitting is a serious problem in such networks. Large networks are also slow to use, making it difficult to deal with overfitting by combining the predictions of many different large neural nets at test time. Dropout is a technique for addressing this problem. The key idea is to randomly drop units (along with their connections) from the neural network during training [43]. Sixty-five percent of connections were randomly kept while training the network. Finally, the last fully-connected layer after the dropout layer gives the prediction for the number of tomatoes in the input image. Batch normalization was performed after every convolution to remove the internal covariate shift [38]. 

### 3.5. Training Methodology

The network was trained for three epochs on 24,000 synthetic images. To minimize the error an Adam optimizer is used [44]. It is an algorithm for first-order gradient-based optimization of stochastic objective functions, based on adaptive estimates of lower-order moments. Inspired by two popular optimized methods, namely, AdaGrad and RMSProp, Kingma and Ba [44] came up with a new optimizer that can deal with a sparse gradient, as well as non-stationary objectives. The advantages of using the Adam optimizer include being computationally efficient, low memory requirements, invariance to diagonal rescaling of the gradients, and being well-suited for problems that are large in terms of data or parameters. The learning rate for the Adam optimizer was set at a constant 1 × 10^−3^. The mean squared error was used as the cost function. The network was evaluated using the exponential moving averages of weights. Weights were initialized using a Xavier initializer [45]. Xavier initialization ensures the weights are appropriate by keeping the signal in a reasonable range of values throughout the layers. It tries to keep the variance of the input gradient and the output gradient the same which helps to keep the scale of the gradients approximately the same throughout the network.

## 4. Experimental Results 

The network was implemented using TensorFlow [46] running on an NVidia 980Ti GPU. For training, 24,000 synthetic images were used. For testing, a different set of 2400 synthetic images and 100 randomly-selected real tomato images from Google Images were used. The size of synthetic images are 128 × 128 pixels. Real images have different sizes, but all were resized to 128 × 128 pixels.

### 4.1. Experimental Results with Synthetic Data

**Figure 6:**
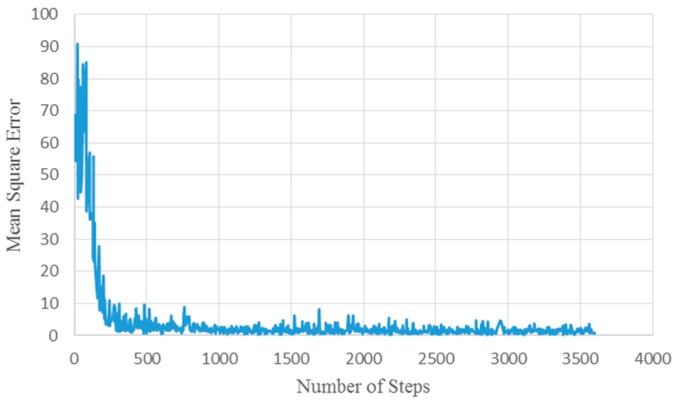
Mean square error for training at a dropout value of 65%.

Validation on 2400 synthetic images gives a mean squared error for the count of about 1.16. Figure 6 shows the mean square error for training where the abscissa represents the number of steps and the ordinate represents the mean square error. The network was trained with three different dropout values (50%, 65%, and 80%) to find the lowest value for the mean square error, and 65% was chosen as the dropout value for the network. Figure 6 shows the mean square error for a dropout value of 65%. Looking at the graph in Figure 6, it is clear that the network converges quickly; this is why the network was trained for only three epochs. 

### 4.2. Experimental Results with Real Data

The algorithm was tested over 100 randomly-chosen images; despite not having real images for training, the network performs well for real images. Table 1 shows twenty representative images along with their predicted and actual count. In Table 1 column R contains the real images, column P contains the predicted count, and column GT contains the actual count (ground truth). 

The network was trained to count ripe and half-ripe tomatoes. The algorithm can handle the variation in illumination, size, shadow, and also images with overlapped and partially-occluded fruits. For example, fruits in the second row and fourth column image are partially occluded by leaves and they are under different illumination conditions. However, the actual count and predicted count by our algorithm are exactly the same (=12). Another example is the image in the last row and the last column, which has overlapped fruits with different sizes and is occluded by foliage; still, the actual count and predicted count by our algorithm are exactly the same (=24). 

### 4.3. Evaluation

To evaluate the performance of our results, we compared the predicted count of our algorithm with the actual count. The actual count was attained by taking the average count of three individuals observing the images independently. 

**Figure 7:**
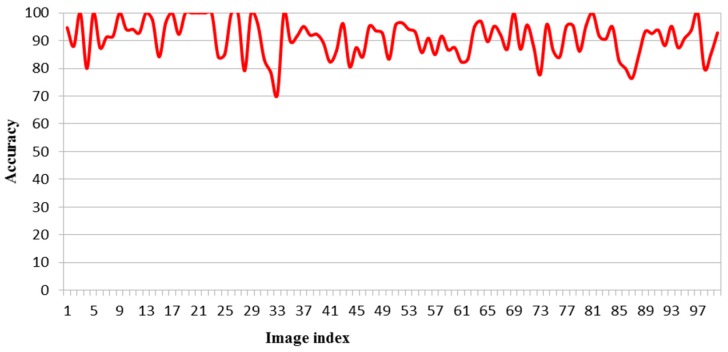
The accuracy for all 100 images.

The accuracy was calculated as follows:

(1) $$pa(\%)=\left[1-\frac{\left|pc-ac\right|}{\left|ac\right|}\right]\times 100$$

where, *pa* is the accuracy (%), *pc* is the predicted count, and *ac* is the actual count. As can be seen in Figure 7, the accuracy is between 70% and 100% and the average accuracy for 100 images is equal to 91.03%. 

**Figure 8:**
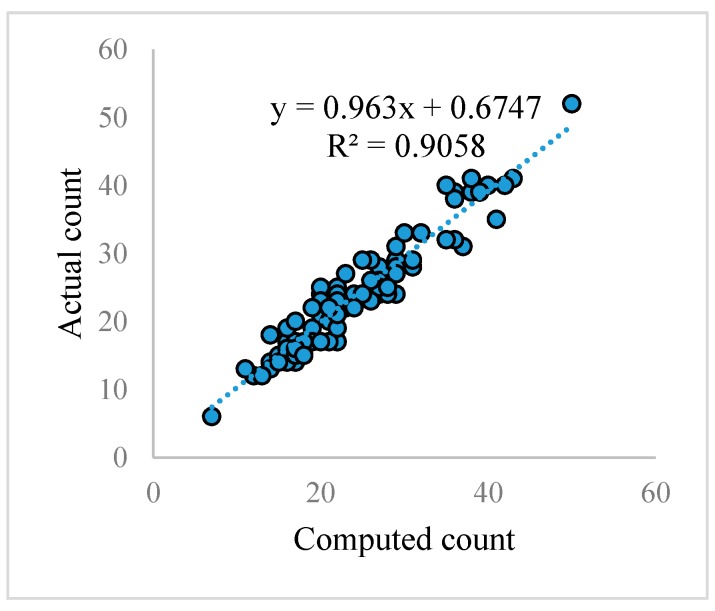
A linear regression between computed and actual counts for 100 real tomato images.

A linear regression was performed between computed and actual counts as shown in Figure 8. *R^2^* value of 0.90 in Figure 8 suggests that the regression line fits well over the data which means the computed count of the tomatoes is similar to the actual count. The root mean square error (RMSE) for 2400 synthetic images is equal to 1.16, and for 100 real images it is 2.52, based on our proposed method.

### 4.4. Comparison with Other Techniques

We compared our results with several methods, namely area-based technique, shallow neural network, and our network with the original Inception-ResNet. 

**Figure 9:**
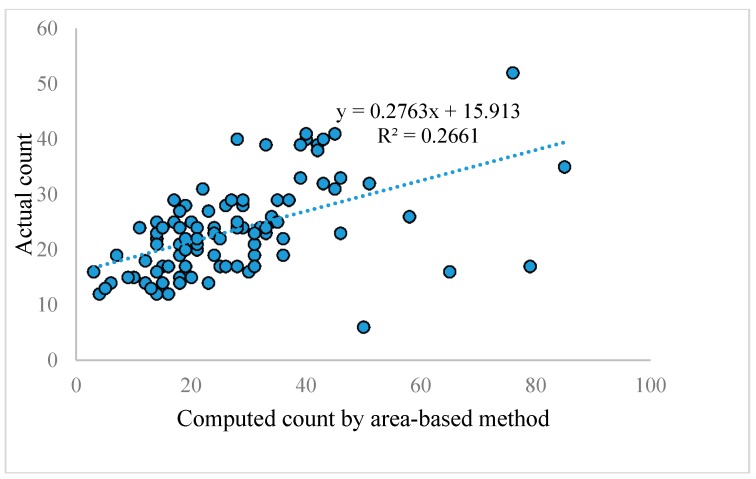
A linear regression between computed counts by the area-based method and the actual count for 100 real tomato images.

Area-based techniques calculate the number of fruits based on the total area of fruits and an individual fruit. We applied mathematical morphology techniques after converting our RGB images to YCBCR space to isolate the pixels that belong to tomatoes. After calculating the total fruit pixels in each image, we divided it by the average pixel coverage of one tomato to get the count. The average pixel number of each tomato was attained experimentally so that there is a minimum distance between the actual count and the calculated count based on the area. The average accuracy over one hundred images for this method is 66.16%. Figure 9 shows a linear regression between computed count by area based method and actual count for one hundred real tomato images. The RMSE of 100 real images based on this method is 13.56. 

It can be inferred from Figure 9 that the performance of the method is not consistent and the *R^2^* value is also very poor. 

We also trained and tested the results over a shallow network. The shallow network consists of two convolutional layers and two fully-connected layers. In the third method, we used the original Inception-ResNet-A module of the Inception-ResNet-v4 [7] layer instead of our modified version in Figure 4. Table 2 shows the average accuracy over one hundred images using the proposed method and three other methods. 

It can be inferred from Table 2 that the proposed method is significantly better than the area-based method. The reason is that the area-based method is not scale invariant. Moreover, the main problem with area-based methods is that whenever there is occlusion by other tomatoes, foliage, or branches, the total pixel coverage of the tomatoes will be less than the actual coverage and will lead to a false count of the tomatoes.

Table 3 shows the average time required counting the tomatoes in one test image using the proposed method, the area-based method, and the time required by a human.

With the help of Table 3, it is clear that the proposed method is faster than that of the area-based and manual counting methods. 

## 5. Conclusion and Future Works

We proposed a simulated learning for counting fruits. We based our architecture on Inception-ResNet to achieve high accuracy and to lower the computation cost. It is very difficult to obtain a sufficient number of real images with their actual count for the training stage in deep learning; in this paper we generated synthetic tomato images for training the network. We observed 91% accuracy for one hundred randomly-chosen real images. Our algorithm is robust under poor conditions. It can count accurately even if tomatoes are under shadow, occluded by foliage, branches, or if there is some degree of overlap amongst tomatoes. Although our algorithm was trained to count tomatoes, it can be applied to other fruits. Our algorithm is able to count ripe and half-ripe fruits; however, it fails to count green fruits because it is not trained for this purpose. In the future, we are planning to add green fruits to the synthetic dataset, so it would be able to count fruits in all stages.

In the future, we are planning to develop a mobile application based on the proposed algorithm which can be used directly by farmers for yield estimation and cultivation practices. Moreover, the proposed algorithm will be implemented on unmanned ground vehicles (UGVs) and unmanned aerial vehicles (UAVs) for online yield estimation and precision agriculture applications. Our efforts will directly support citizen science. 

## Figures and Tables

**Table 1:** Real tomato images with predicted (P) and actual count (GT).

R	P	GT	R	P	GT	R	P	GT	R	P	GT
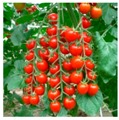	36	38	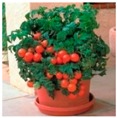	27	24	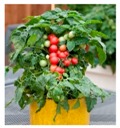	18	17	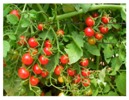	27	28
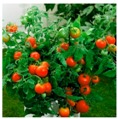	22	25	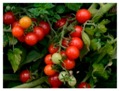	21	23	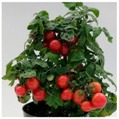	15	14	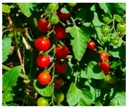	12	12
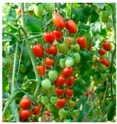	22	22	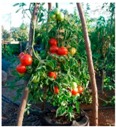	13	12	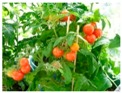	14	14	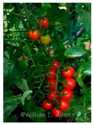	14	13
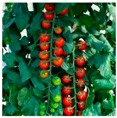	20	25	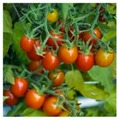	19	19	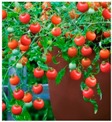	38	39	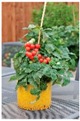	16	16
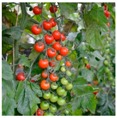	22	22	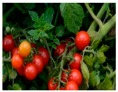	16	17	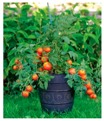	16	19	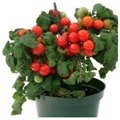	24	24

**Table 2:** Average accuracy over 100 images.

Method	Average Accuracy (%)
Proposed method	91.03
Area-based counting	66.16
Shallow network	11.60
Our network with the original Inception-ResNet-A	76.00

**Table 3:** Average time for counting.

Method	Average Time Required for One Test Image (second)
Proposed method	0.006
Area-based method	0.05
Manual counting	6.5
